# Challenges in Implementing an Explicit Protocol for Live Discharge From Hospice

**DOI:** 10.1093/geroni/igab046.238

**Published:** 2021-12-17

**Authors:** Stephanie Wladkowski, Susan Enguidanos, Tracy Schroepfer, Keegan Pabst

**Affiliations:** 1 Eastern Michigan University School of Social Work, Eastern Michigan University, Michigan, United States; 2 University of Southern California, Los Angeles, California, United States; 3 University of Wisconsin-Madison, Madison, Wisconsin, United States; 4 Eastern Michigan University, Ypsilanti, Michigan, United States

## Abstract

A live discharge from hospice can occur when a patient stabilized under hospice care and no longer meets the life expectancy hospice eligibility criteria. In 2018, 220,000 hospice patients across the United States were discharged alive from hospice care, with 1 in 6 discharges due to stabilization, with a life expectancy exceeding hospice’s six-month criteria. Hospice practitioners prepare patients and their caregivers upon enrollment for the possibility of a live discharge should their condition stabilize, however, there is no explicit discharge process available within hospice to guide practitioners in transitioning patients (and caregivers) out of hospice care. This transition process largely falls within the domain of hospice social workers, yet there is no research documenting the challenges and facilitators to conducting a live discharge from hospice. This study aimed to understand social workers' perspectives on the live discharge process. To better understand challenges and facilitators to the live discharge process, we conducted focus group interviews with hospice social workers at four hospice agencies across the U.S. We asked participants to discuss specific tasks associated with the live discharge process for a patient and their caregiver including identifying concrete services needed post-discharge; assessing the psychosocial and grief risk of patient and caregiver; and developing a post-discharge follow-up plan. Using constant comparison analysis we identified several themes including the need for clear professional roles during a live discharge, interprofessional education, and the need for dedicated time for live discharge follow-up. Policy implications and opportunities also will be discussed.

